# An amino-substituted 2-(2′-hydroxyphenyl)benzimidazole for the fluorescent detection of phosgene based on an ESIPT mechanism†

**DOI:** 10.1039/d1ra00811k

**Published:** 2021-03-15

**Authors:** Zi-Jie Li, Wen-Jie Zhang, Wen-Zhu Bi, Qiu-Juan Ma, Su-Xiang Feng, Xiao-Lan Chen, Ling-Bo Qu

**Affiliations:** School of Pharmacy, Henan University of Chinese Medicine Zhengzhou 450046 China biwenzhu2018@hactcm.edu.cn +86 371 65962746 +86 371 65962746; Collaborative Innovation Center for Respiratory Disease Diagnosis and Treatment & Chinese Medicine Development of Henan Province Zhengzhou 450046 China; Zhengzhou Key Laboratory of Chinese Medicine Quality Control and Evaluation Zhengzhou 450046 China; College of Chemistry, Zhengzhou University Zhengzhou 450052 China

## Abstract

In this work, an ESIPT-based fluorescence probe, 5′-amino-2-(2′-hydroxyphenyl)benzimidazole (P1), was synthesized and explored for the ratiometric detection of phosgene. Compared to 2-(2′-hydroxyphenyl)benzimidazole (HBI), P1 exhibits high sensitivity (LoD = 5.3 nM) and selectivity toward phosgene with the introduction of the amine group. Furthermore, simple P1 loaded test papers are manufactured and display selective fluorescent detection of phosgene in the gas phase.

## Abstract 2

An easily prepared phosgene probe, 5′-amino-2-(2′-hydroxyphenyl)benzimidazole (P1), is designed and studied. Based on ESIPT mechanism, P1 exhibits ratiometric, sensitive and selective detection of phosgene both in solution and gas phase.

## Graphical abstract

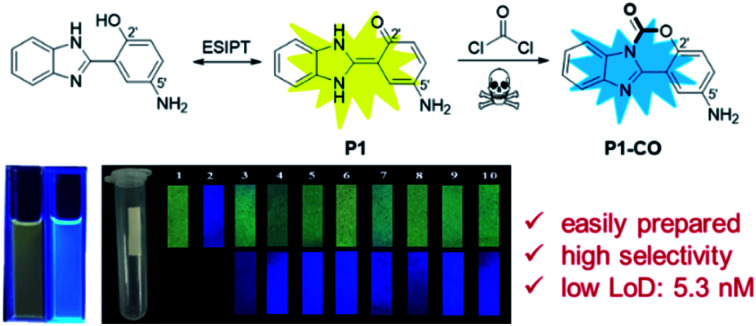

## Introduction

Phosgene (COCl_2_), also called carbonyl chloride, is a kind of colorless gas with a freshly cut grass-like odor and is widely used as an important industrial ingredient for the production of pesticides, pharmaceuticals, dyes, numerous chemicals and materials.^1–6^ On the other hand, due to its fast reaction with alveoli protein, disrupting the blood-air barrier and resulting in suffocation,^7–9^ phosgene is notorious for its abuse as a chemical weapon during World War I.^10–14^ However, unlike other chemical weapons such as sarin, tabun, soman and VX, prohibited by laws all over the world,^15^ phosgene can be easily generated by readily available triphosgene in the presence of tertiary amines or chloride ions.^2,16^ The extensive industrial use, high toxicity and easy accessibility make phosgene a serious threat to public health and safety. Therefore, the development of facile and effective strategies for its rapid detection are urgent and necessary.

As to the detection of phosgene, conventional gas chromatography^17–19^ and electrochemical technique^20^ suffer from obvious drawbacks, such as bad portability, high cost and inconvenience in real-time detection. Nowadays, fluorescent detection methods are well developed and widespread due to their easy manipulation, fast response, high sensitivity and selectivity, and the possibility of real-time detection. Among them, reports on the fluorescent detection of phosgene are still limited^12–14^ and mainly based on: (i) twice carbamylation reactions of fluorescence probes, which employ *o*-phenylenediamine,^21^*o*-hydroxyaniline,^22^*o*-aminobenzyl amine,^23^ catechol,^24^ ethylenediamine,^25^ ethanolamine^26^ or other moieties^27^ as reactive site; (ii) phosgene-promoted dehydration reaction of fluorescence probes, which employ oxime^28^ or amide^29^ as reactive site; and (iii) several other phosgene-induced reactions, including intermolecular reaction of two fluorophores,^30^ intramolecular reaction of cinnamic acids,^31^ ring opening reaction of benzimidazole-fused rhodamine dye^32^ and Beckmann rearrangement of ketoxime^33^ (Table S2†). However, most of these fluorescence probes are based on mechanisms of photoinduced electron transfer (PeT), intramolecular charge transfer (ICT) or aggregation-induced emission (AIE), which might be disturbed by acetylating,^21*c*^ phosphorylating agents^21*h*^ or oxidizing chemicals,^21*e*,27*g*^ resulting to false response. And several other drawbacks still need to be overcome, such as incapable of discrimination between triphosgene and phosgene^21*b*,21*f*^ and tedious preparation process.^22,24,25^

Due to the promising advantages, such as high selectivity, high sensitivity and large Stokes shift, excited-state intramolecular proton transfer (ESIPT) based fluorophores has attracted considerable attention in the development of new fluorescence detection methods.^34^ However, only two ESIPT based fluorescent probes have been developed for the ratiometric detection of phosgene through twice carbamylation of their keto or enol from, *i.e.* 2-(2-aminophenyl)benzothiazole (ABT) reported by Chen *et al.* in 2017^35^ and 2-(1*H*-phenanthro[9,10-*d*]imidazol-2-yl)phenol (Pi) reported by Wu *et al.* in 2019.^36^ Despite successful application of the two ESIPT based probes, ABT is unable to discriminate between phosgene and triphosgene, and Pi suffers from high LoD in solution (0.14 μM).

**Scheme 1:**
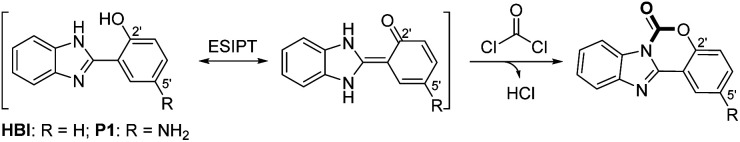
The design strategy of ESIPT-based fluorescence probes for phosgene.

In order to overcome these drawbacks and develop ‘6S’ (simpleness, speedy, selectivity, sensitivity, stability and smart) fluorescence probe for the detection of phosgene, we select a readily prepared small molecule, 2-(2′-hydroxyphenyl)benzimidazole (HBI), as our initially explored fluorophore (Scheme 1, R = H). Preliminary experiments in our group showed a fast and sensitive reaction between HBI and phosgene at a concentration of 10 μM, resulting to an obvious fluorescent intensity increase at 351 nm and decrease at 462 nm. However, no obvious color change was observed under hand-held 365 nm UV light and the selectivity of HBI was not satisfactory (Fig. S1†). Inspired by the fast and sensitive reaction of HBI toward phosgene, we continuously committed our efforts to develop new HBI derivatives with large Stokes shifted emission and high selectivity. Herein, we report an amino modified 2-(2′-hydroxyphenyl)benzimidazole (P1) for the ratiometric detection of phosgene (Scheme 1, R = NH_2_). Through the introducing of the strong electron-donating amino group at the C5′ position of HBI, the fluorescent emission is red-shifted to 540 nm. Upon addition of phosgene, P1 is converted to a six-membered ring-containing product P1-CO, the ESIPT process is disrupted and the emission color changes from yellow to bright blue under hand-held 365 nm UV light. Moreover, compared to HBI, the selectivity of P1 is also well improved. Furthermore, simple and easily prepared P1 loaded test papers are fabricated and display an obvious fluorescence color change upon exposure to phosgene in the gas phase.

## Experimental section

### Reagents and instruments

All reagents and solvents were obtained from commercial suppliers and used without further purification. ^1^H and ^13^C NMR spectra were recorded on a Bruker 400 spectrometer in DMSO-d_6_ containing tetramethylsilane as an internal standard. Fluorescence emission spectra were collected by Hitachi F7000 fluorescence spectrometer. UV-Vis absorption spectroscopy measurements were performed on Thermo Evolution 260 Bio at room temperature. High-resolution mass spectra (HRMS) were obtained with a Thermo LTQ Orbitrap mass spectrometer.

### Synthesis and characterization of P1

5-Aminosalicylic acid (0.153 g, 1.0 mmol) and *o*-phenylenediamine (0.108 g, 1.0 mmol) were mixed and stirred in polyphosphoric acid (85% phosphorus pentoxide, 10 mL) at 160 °C over a period of 3 h.^37^ Then the reaction mixture was poured into distilled water (20 mL), and the precipitate were collected and washed by saturated NaHCO_3_ aqueous solution to pH 8–9. The obtained crude product was further purified by column chromatography (ethyl acetate: petroleum ether = 3 : 1) to give P1 (0.108 g, yield 48%) as a purple solid. ^1^H NMR (400 MHz, DMSO-d_6_) *δ* (ppm): 13.01 (br, s, 1H, NH), 12.16 (br, s, 1H, OH), 7.68–7.59 (m, 2H, Ar-H), 7.26–7.23 (m, 3H, Ar-H), 6.79 (d, *J* = 8.0 Hz, 1H, Ar-H), 6.72 (dd, *J* = 2.2 Hz, *J* = 7.1 Hz, 1H, Ar-H), 4.70 (br, s, 2H, NH_2_); ^13^C NMR (100 MHz, DMSO-d_6_) *δ* (ppm): 152.59, 149.93, 141.28, 119.68, 117.93, 112.80, 110.89. HRMS: [M + H]^+^: calcd for C_13_H_12_N_3_O: 226.0975, found: 226.0980.

### Synthesis and characterization of P1-CO

P1 (0.113 g, 0.5 mmol) and triethylamine (0.1 mL) were dissolved in dry CH_2_Cl_2_ (15 mL) at 0 °C, then triphosgene (0.15 g, 0.5 mmol) in dry CH_2_Cl_2_ (5 mL) was added over a period of 10 min. The mixture was continually stirred at 0 °C until the completion of the reaction. Saturated NaHCO_3_ aqueous solution was added into the mixture and extracted with CH_2_Cl_2_ (20 mL × 2). The organic phase was collected, dried over anhydrous Na_2_SO_4_ and evaporated to give the crude product. The crude product was further purified by column chromatography (ethyl acetate: petroleum ether = 1 : 2) to give P1-CO (0.098 g, yield 80%) as a brown solid. ^1^H NMR (400 MHz, DMSO-d_6_) *δ* (ppm): 8.18–8.16 (m, 1H, Ar-H), 7.86–7.84 (m, 1H, Ar-H), 7.51–7.48 (m, 2H, Ar-H), 7.39 (d, *J* = 2.2 Hz, 1H, Ar-H), 7.28 (d, *J* = 7.1 Hz, 1H, Ar-H), 6.95 (dd, *J* = 2.2 Hz, *J* = 7.1 Hz 1H, Ar-H), 5.56 (br, s, 2H, NH_2_); ^13^C NMR (100 MHz, DMSO-d_6_) *δ* (ppm): 147.44, 147.23, 143.98, 143.83, 142.99, 130.99, 126.07, 125.15, 120.03, 119.88, 117.76, 114.78, 112.98, 106.60. HRMS: [M + H]^+^: calcd for C_14_H_10_N_3_O: 252.0768, found: 252.0770.

### General analytical experiment procedure

Phosgene was generated by addition of the less-toxic triphosgene to the solution of TEA (triethylamine). Stock solutions of P1 and triphosgene for UV-Vis and fluorescence titrations were prepared in CH_2_Cl_2_ (1.0 mM). Stock solution of triethylamine (TEA) was prepared in CH_2_Cl_2_ (0.1 vol%). Relevant analytes ((COCl)_2_, CH_3_COCl, SOCl_2_, TsCl, DCP, HOAc, POCl_3_ and SO_2_Cl_2_) solutions were also prepared in CH_2_Cl_2_ (10 mM). The test solutions were prepared by dilution of the stock solutions. For detection of phosgene in solutions: 20 μL probe solution and equal amount of TEA solution were added and mixed in 2.0 mL of CH_2_Cl_2_, then triphosgene stock solution (1–12 μL) was added to generate phosgene. For detection of relevant analytes in solutions: 20 μL probe solutions and equal amount of TEA solution were added and mixed in 2.0 mL of CH_2_Cl_2_ and then 10 μL relevant analytes stock solutions were added. All solutions were kept for 10 min before the UV-Vis and fluorescence testing.

### Preparation of P1 loaded test papers and detection of gaseous phosgene

Filter paper was cut into small strips (0.5 cm × 2.5 cm) and dipped in the P1 stock solution (1.0 mM), then removed and dried under air. The dip-dry process was repeated for 2 times. For detection of gaseous phosgene: the pre-prepared filter paper was firstly pasted on the inner wall of a 10 mL centrifuge tube, and 20 μL TEA stock solution (0.1 vol% in CH_2_Cl_2_) was added by the use of a HPLC needle. Following that, 2 μL, 4 μL, 8 μL, 12 μL and 16 μL of triphosgene solution (10 mM in CH_2_Cl_2_) were added in the centrifugal tube, respectively. Thus, the concentration of phosgene was calculated to be 0.6 mg L^−1^, 1.2 mg L^−1^, 2.4 mg L^−1^, 3.6 mg L^−1^ and 4.8 mg L^−1^, assuming that triphosgene was completely decomposed into gaseous phosgene. The centrifugal tubes were sealed immediately and kept for 10 min, then imaged under 365 nm UV-light. For detection of other gaseous analytes: (COCl)_2_, CH_3_COCl, SOCl_2_, TsCl, DCP, HOAc, POCl_3_ and SO_2_Cl_2_ (4 μL, 0.1 M in CH_2_Cl_2_) were added in the above mentioned 10 mL centrifugal tube with our pre-prepared filter paper. Then, the centrifugal tubes were sealed and kept for 10 min. The fluorescent response to each analytes vapor was observed and imaged under 365 nm UV-light, respectively.

## Results and discussion

### Synthetic process of P1 and P1-CO

**Scheme 2:**
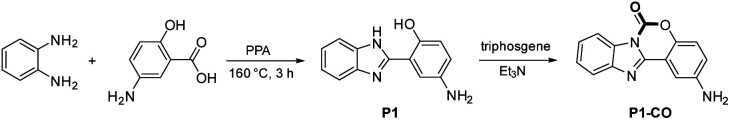
Synthesis of P1 and P1-CO.

As shown in Scheme 2, P1 was synthesized by one-pot condensation reaction of *o*-phenylenediamine with 5-aminosalicylic acid in PPA at 160 °C for 3 h.^37^ The related proposed sensing product P1-CO was synthesized through fast twice carbamylation reaction between P1 and triphosgene in the presence of TEA with good isolated yield. HPLC-MS analysis of the reaction mixture showed a complete conversion of P1 and the 5′-amino group did not react with phosgene under this condition (Fig. S7†). The structures of P1 and P1-CO were fully characterized by ^1^H NMR, ^13^C NMR and high-resolution mass spectroscopy (HRMS).

### Photo physics property of P1 and P1-CO

**Fig. 1:**
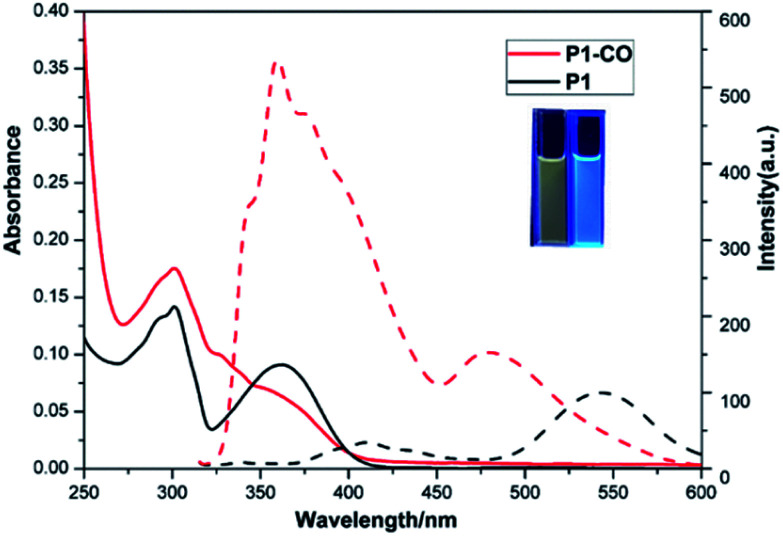
UV-Vis absorption (solid) and fluorescence (dash) spectra of 10 μM P1 (black) and 10 μM P1-CO (red) in CH_2_Cl_2_ solutions. Inset: Photos of P1 (left) and P1-CO (right) solutions under 365 nm UV-light.

As shown in Fig. 1, the photo physics property of the as-synthetic two compounds were investigated. Due to the intramolecular proton transfer of the hydroxyl and imidazole moieties, P1 in CH_2_Cl_2_ (10 μM) displays the absorption spectral maximum (*λ*^abs^_max_) at 305 and 355 nm and a mainly yellow fluorescent emission maximum (*λ*^em^_max_) at 540 nm. While, the absorption and fluorescence spectral maximum (*λ*^abs^_max_/*λ*^em^_max_) of P1-CO were observed to be 305/358 nm with a blue fluorescent emission. The fluorescence quantum yields (*Φ*_f_) of P1 (4.3%) and P1-CO (56%) were measured with quinine sulfate as the reference (*Φ*_f_ = 54% in 1.0 M H_2_SO_4_) (Fig. S2†). Therefore, P1 would be employed as a ratiometric fluorescent probe for detection of phosgene with a fluorescence-emission color change from yellow to blue under 365 nm UV lamp (Fig. 1, inset).

### Fluorescence titration of P1 to phosgene

**Fig. 2:**
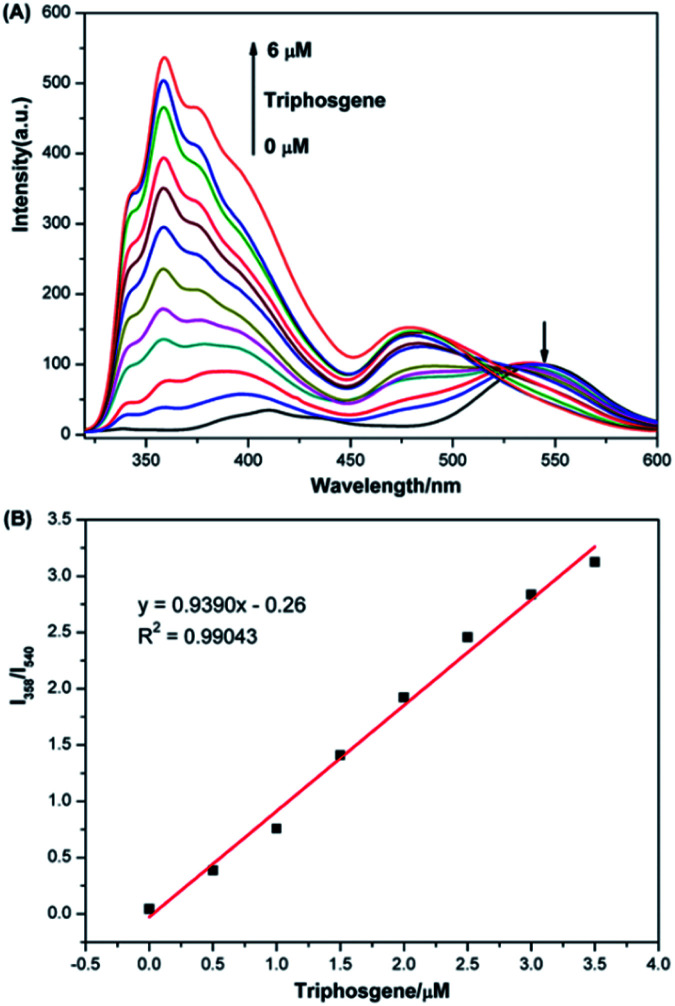
(A) Fluorescence spectra of 10 μM P1 solutions containing TEA (20 μL, 0.1 vol%) upon addition of triphosgene (0–6 μM), *λ*_ex_ = 305 nm, slit width = 5/5 nm. (B) Linear correlation between the ratio of emission intensities at 358 nm and 540 nm (*I*_358_/*I*_540_) and the concentration of triphosgene (0–3.5 μM).

The fluorescence titration analysis of P1 to phosgene was performed as shown in Fig. 2A. The fluorescent spectra of P1 showed a regular change after 0–6 μM triphosgene was added. The emission intensity at 540 nm gradually decreased while the peak at 358 nm increased. The ratio of the emission intensities at 358 and 540 nm was found to depend linearly on the triphosgene concentration over the 0–3.5 μM range (Fig. 2B). The triphosgene detection limit was determined to be 5.3 nM (LoD = 3*σ*/*k*, where *σ* is the standard deviation of the blank experiment, and *k* is the slope of the relationship between the emission-intensity ratio and the phosgene concentration) (Fig. S4†).

### Response time of P1 to phosgene

**Fig. 3:**
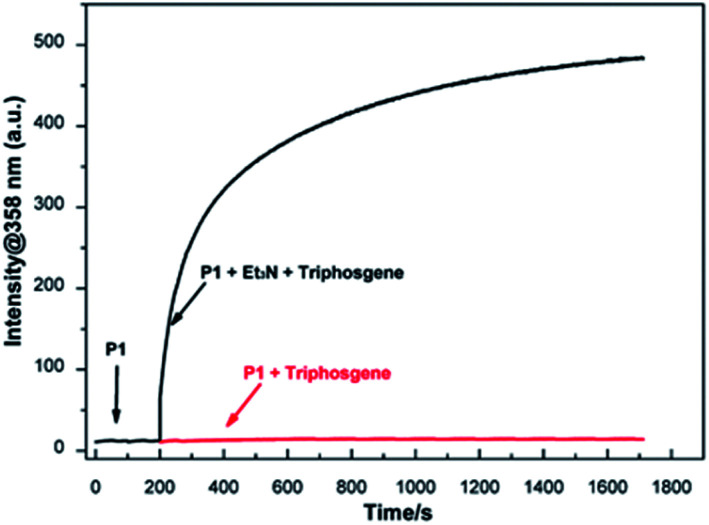
Time-dependent fluorescence intensity at 358 nm of P1 (10 μM) treated with triphosgene (6 μM) at 200 s in the presence (black line) or absence (red line) of TEA, *λ*_ex_ = 305 nm, *λ*_em_ = 358 nm, slit width = 5/5 nm.

Time-dependent fluorescence spectral was performed to assess the response time of P1 to phosgene. As shown in Fig. 3, the fluorescence intensities at 358 nm for 10 μM P1 solution with 6 μM triphosgene in the presence (black line) or absence (red line) of TEA (20 μL, 0.1 vol%) was recorded. As it can be seen, the fluorescence intensity at 358 nm rapidly increased (within 50 s) upon the addition of triphosgene and TEA (black line). On the contrary, there is no obvious change in the fluorescence intensity at 358 nm without TEA (red line). Therefore, due to the different reactivity of P1 to phosgene and triphosgene, the designed probe P1 can efficiently discriminate between phosgene and triphosgene.

### Sensing mechanism of P1 to phosgene

**Fig. 4:**
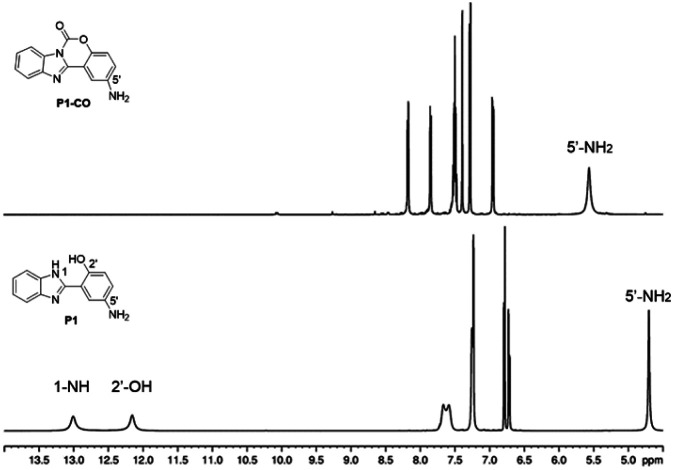
^1^H NMR spectra of P1 and P1-CO.

Initially, P1 displayed dual fluorescence emission at 412 nm and the mainly 540 nm (Fig. 1), which should be the sign of ESIPT and indicated the coexistence of the keto and enol form coexist of P1 in solution.^38^ Upon addition of phosgene, the hydroxyl and imidazole moieties of P1 were connected by the carbonyl group, resulting to an intramolecular six-membered ring. The keto–enol tautomerization and the ESIPT process of P1 was blocked. The bluish fluorescent emission of P1-CO could be attributed to the ESIPT ‘enol’ form. In order to confirm the proposed mechanism, P1 and the proposed sensing product P1-CO were characterized by ^1^H NMR. As shown in Fig. 4, the peaks at 12.16 ppm for hydroxyl and 13.01 ppm for the imidazole amino groups disappear, which confirms that these groups serve as the phosgene-recognition sites. Moreover, the proposed sensing product was characterized by HRMS for C_14_H_10_N_3_O: M + H^+^: calculated 252.0768, found 252.0770 (Fig. S13†), indicating that the molecular weight (*m*/*z*) of the purified product corresponds with that of P1-CO. These data strongly backed the proposed mechanism.

### Sensing selectivity of P1 to phosgene

**Fig. 5:**
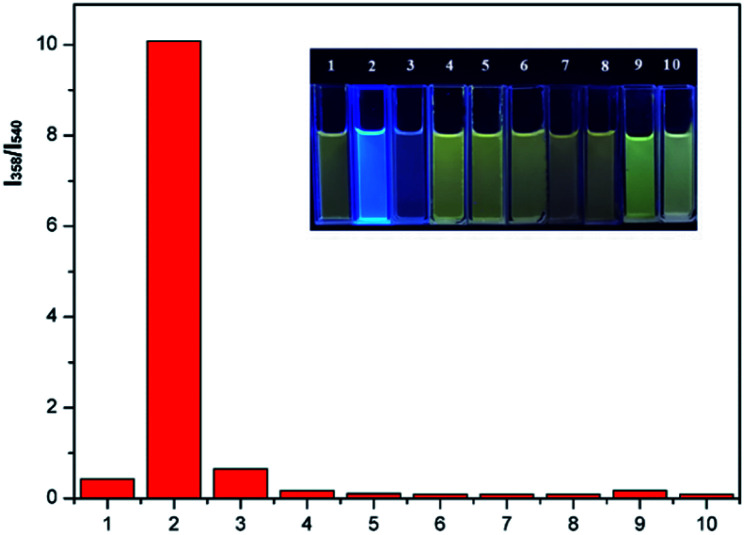
Fluorescence increments at 358 nm and 540 nm relative to 10 μM P1 solutions containing TEA (20 μL, 0.1 vol%) before and after addition of triphosgene (6 μM) or various analytes (50 μM) for 10 min: (1) blank, (2) triphosgene, (3) (COCl)_2_, (4) CH_3_COCl, (5) SOCl_2_, (6) TsCl, (7) DCP, (8) HOAc, (9) POCl_3_, (10) SO_2_Cl_2_. *λ*_ex_ = 305 nm. Inset: Photo of above solutions under 365 nm UV-light.

The selectivity of P1 for the detection of phosgene over various potential interferents were assessed. As shown in Fig. 5, 10 μM P1 solutions containing TEA (20 μL, 0.1 vol%) were treated with phosgene (triphosgene (6 μM)) and 50 μM interferents solutions. The fluorescence spectroscopy reveals a clear change for phosgene at 358 nm (Fig. S5†). The fluorescence increments at 358 nm and 540 nm exhibits a highly specific and selective detection of phosgene over various acyl chlorides. Moreover, the selectivity of P1 can be obviously observed under 365 nm UV-light from yellow to blue only for phosgene (Fig. 5, inset). Furthermore, the determination of phosgene in the presence of these interfering compounds were performed. As shown in Fig. S6 and Table S1,† the fluorescent intensities at 358 nm were not significantly affected by these interfering compounds at 5.0 μM and good recovery rate was obtained (from 87.8% to 108.9%). However, increased concentration (10 μM) would cause obvious fluorescent intensity decrease. This might be due to the block of recognition sites by interfering compounds at high concentration.

### Detection of gaseous phosgene and other vapors

**Fig. 6:**
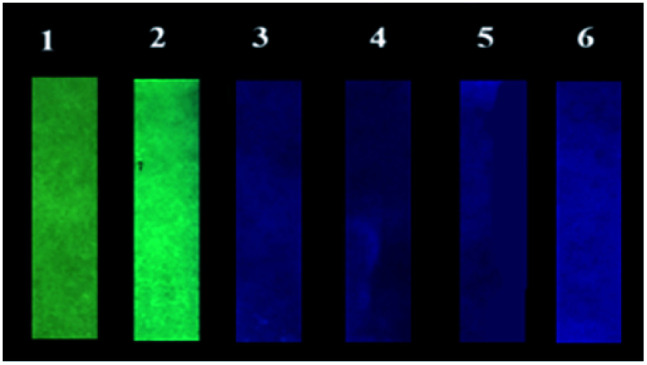
Photographic images of P1 test papers upon exposure to various amounts of gaseous phosgene generated by triphosgene and TEA in 10 mL centrifugal tubes. (1) 0 mg L^−1^, (2) 0.6 mg L^−1^, (3) 1.2 mg L^−1^, (4) 2.4 mg L^−1^, (5) 3.6 mg L^−1^, (6) 4.8 mg L^−1^.

**Fig. 7:**
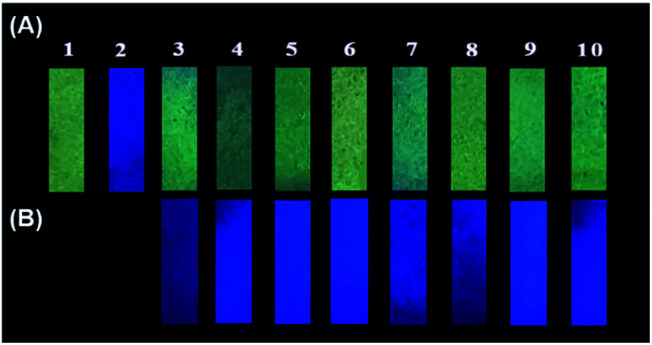
Photographic images of P1 test papers upon exposure to (A) phosgene gas (1.2 mg L^−1^) and the vapors of potential interferents (4 μL, 0.1 M in CH_2_Cl_2_) and (B) phosgene gas (1.2 mg L^−1^) after exposure to the vapors of potential interferents. (1) Blank, (2) phosgene, (3) (COCl)_2_, (4) CH_3_COCl, (5) SOCl_2_, (6) TsCl, (7) DCP, (8) HOAc, (9) POCl_3_, (10) SO_2_Cl_2_.

In order to investigate the possible application of P1 for the detection of gaseous phosgene, simple and low cost P1 test papers were prepared. As shown in Fig. 6, P1 test paper exhibited yellow fluorescence under 365 nm UV light. After exposure to various amounts of gaseous phosgene (0–4.8 mg L^−1^), a yellow-green-blue fluorescence color change was clearly observed. Moreover, the selectivity of P1 loaded test paper to phosgene over other related analytes were also investigated (Fig. 7). The results showed that P1 test paper did not give false responses to other potential interferents. More importantly, the detection of gaseous phosgene were also founded effective even though the test paper was pre-exposed to the vapors of potential interferents. Therefore, based on all these results, P1 can be employed for selective detection of phosgene both in solutions and in gas phase.

## Conclusions

In summary, we have designed and synthesized a new 5′-amino-2-(2′-hydroxyphenyl)benzimidazole (P1) for the fluorescent detection of phosgene both in solution and in gas phase. This probe employs hydroxyl and imidazole moieties as recognition sites to trap phosgene and bears a strong electron donating amino group to expand the Stokes shifted emission and improve the selectivity. Through twice carbamylation of the recognition sites, the ESIPT process of P1 is forbidden and an obvious fluorescent color change from yellow to blue can be easily observed by naked eyes. This probe is sensitive (LoD = 5.3 nM) and highly selective toward phosgene over triphosgene and other acyl chlorides. Furthermore, P1 loaded test paper was fabricated and successfully applied for selective detection of gaseous phosgene with an easily observed yellow to blue color change.

## Author contributions

Zi-Jie Li and Wen-Jie Zhang: methodology, formal analysis, investigation, visualization. Wen-Zhu Bi: conceptualization, analysis, writing-review & editing. Qiu-Juan Ma and Su-Xiang Feng: writing – review & editing, validation. Xiao-Lan Chen and Ling-Bo Qu: validation, supervision.

## Conflicts of interest

There are no conflicts to declare.

## Supplementary Material

RA-011-D1RA00811K-s001
