# Supplementary material for: An amino-substituted 2-(2′-hydroxyphenyl)benzimidazole for the fluorescent detection of phosgene based on an ESIPT mechanism
Source: RSC Adv. 2021 Mar 15;11(18):10836–41. doi: 10.1039/d1ra00811k (PMC8695812; doi:10.1039/d1ra00811k)
Supplement: RA-011-D1RA00811K-s001 [file RA-011-D1RA00811K-s001.pdf]

Electronic Supplementary Material (ESI) for RSC Advances.
This journal is © The Royal Society of Chemistry 2021

Supplementary Information

An amino-substituted 2-(2'-hydroxyphenyl)benzimidazole for the fluorescent detection of phosgene based on ESIPT mechanism

Zi-Jie Li,[a] Wen-Jie Zhang,[a] Wen-Zhu Bi,*[a] Qiu-Juan Ma,*[a] Su-Xiang Feng,*[abc] Xiao-Lan Chen,[d] and Ling-Bo Qu[d]

[a] School of Pharmacy, Henan University of Chinese Medicine, Zhengzhou, 450046, China.

[b] Collaborative Innovation Center for Respiratory Disease Diagnosis and Treatment & Chinese Medicine Development of Henan Province, Zhengzhou, 450046, China

[c] Zhengzhou Key Laboratory of Chinese Medicine Quality Control and Evaluation, Zhengzhou, 450046, China

[d] College of Chemistry, Zhengzhou University, Zhengzhou, 450052, China.

Corresponding E-mail: biwenzhu2018@hactcm.edu.cn

Contents

1. **Evaluation of HBI as the fluorescent probe for the detection of phosgene……....S2**
2. **Measurement of the fluorescence quantum yields..…………………...................…S3**
3. **UV-Vis spectra of P1 upon addition of phosgene……………..……..……..……S4**
4. **Measurement of the LoD for P1……………………………………………….…....…S4**
5. **The selectivity of P1 for the detection of phosgene………………………….…....…S5**
6. **Fluorescence spectra of P1 in the presence of interfering compounds……..…....S6**
7. **Table S1 Determination of phosgene in the presence of interfering compounds..S6**
8. **HPLC-MS chromatogram and spectrum of the reaction mixture………………...….S6**
9. **^{1}H NMR, ^{13}C NMR and HRMS copies of P1 and P1-CO…………………...…..…...…S7**
10. **Table S2 Comparison of recently reported probes for phosgene detection..….....S11**

1. Evaluation of HBI as the fluorescence probe for the detection of phosgene

HBI (2-(2′-hydroxyphenyl)benzimidazole) was synthesized according to literature methods with minor modifications.[1] The method for the evaluation of HBI as the fluorescent

probe for the detection of phosgene was same as that of **P1**.

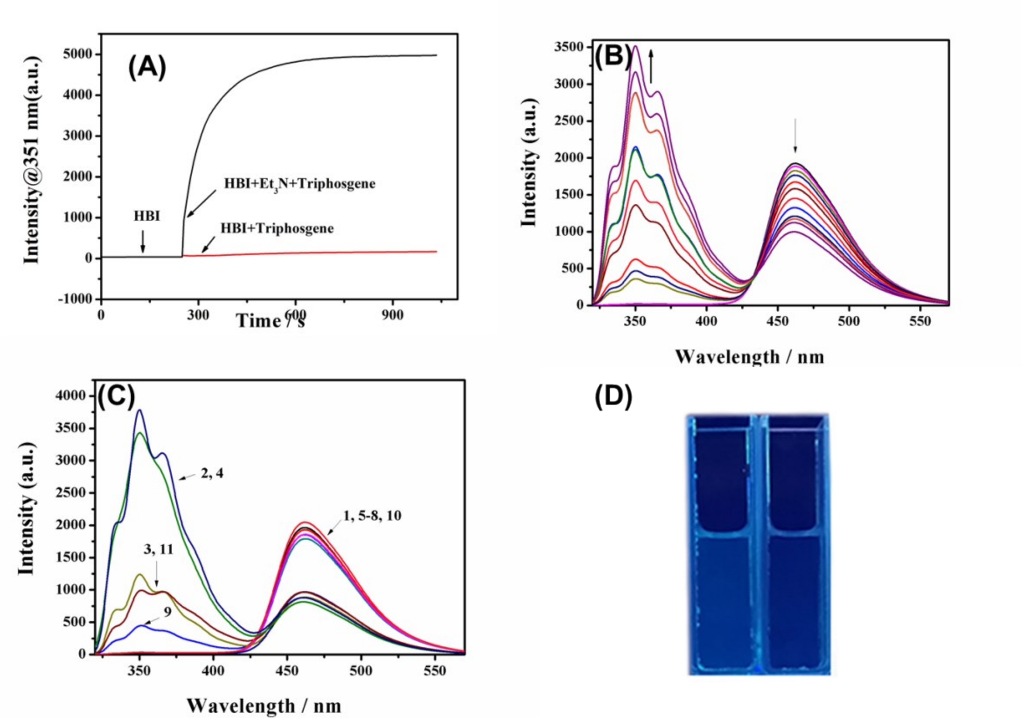

Figure S1 (A) Time-dependent fluorescence intensity at 351 nm of HBI (10 μM) treated with triphosgene (10 μM) in the presence (black line) or absence (red line) of TEA (20 μL, 0.1 vol%), λex = 309 nm, λem = 351 nm, slit width =5/5 nm. (B) Fluorescence spectra of 10 μM HBI solutions containing TEA (20 μL, 0.1 vol%) upon addition of triphosgene (0-5 μM), λex = 309 nm, slit width = 5/5 nm. (C) Fluorescence spectra of HBI solutions before and after addition of phosgene or various analytes (0.2 mM) for 10 min: (1) blank, (2) triphosgene (5 μM)/TEA (20 μL, 0.1 vol%), (3) $(COCl)_2$, (4) CH_3COCl, (5) $SOCl_2$, (6) TsCl, (7) DCP, (8) HOAc, (9) $POCl_3$, (10) SO_2Cl_2, (11) triphosgene. λex = 309 nm. (D) Images of HBI solution before (left) and after (right) addition of phosgene under 365 nm UV-light.

Reference

1. K. Akutsu, S. Mori, K. Shinmei, H. Iwase, Y. Nakano and Y. Fujii, *Talanta*, 2016, **146**, 575.

2. Measurement of the fluorescence quantum yields

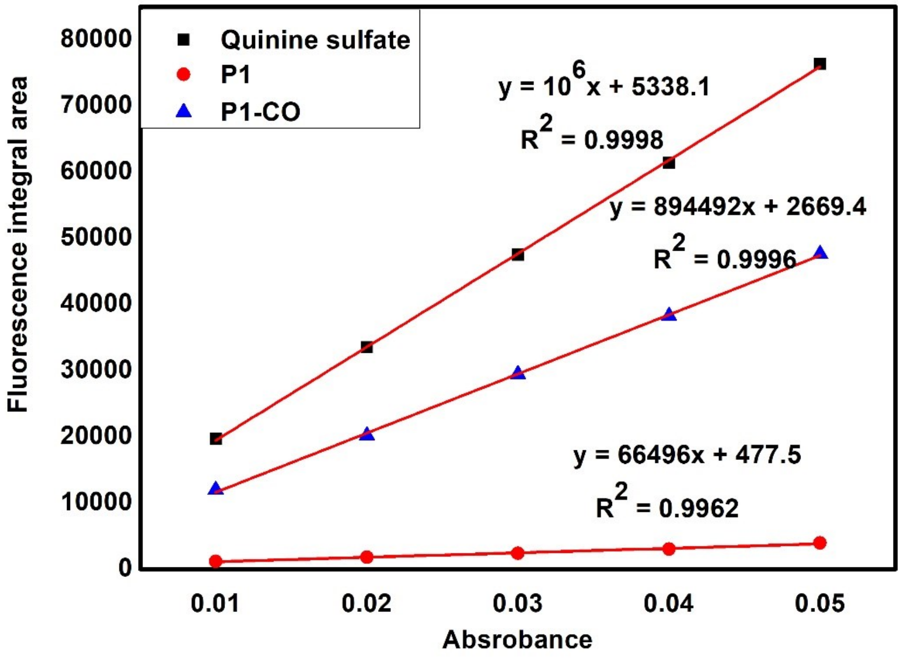

Figure S2 Measurement of the fluorescence quantum yields (Φf) of **P1** and **P1-CO**. **P1** and **P1-CO** were determined in CH_2Cl_2 with solvent refractive index correction. Quinine sulfate in 1.0 M H_2SO_4 was used as the reference (Φf = 54%) at an excitation wavelength of 346 nm. The fluorescence quantum yield was calculated by the following equation: $\Phi_x = \Phi_s (F_x/F_s)(A_s/A_x)(n_x/n_s)^2$. Where x and s indicate the determined and reference, respectively, F is the area of the fluorescence peak, A is the optical density at the excitation wavelength, and n is the refractive index of the solvent.

3. UV-Vis spectra of P1 upon addition of phosgene

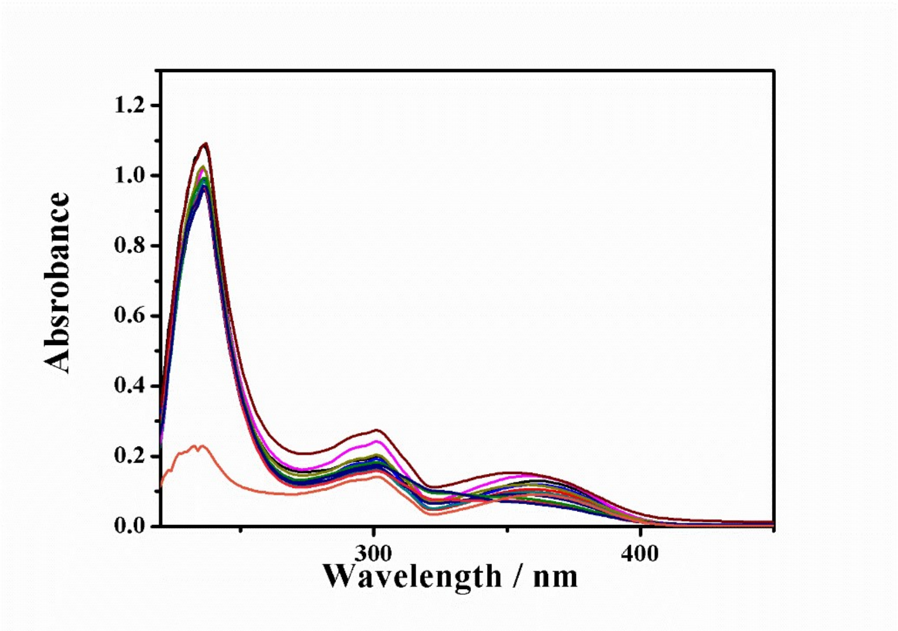

Figure S3. UV-Vis spectra of 10 μM **P1** solutions containing TEA (20 μL, 0.1 vol%) upon addition of triphosgene (0-6 μM).

4. Measurement of the LoD for P1

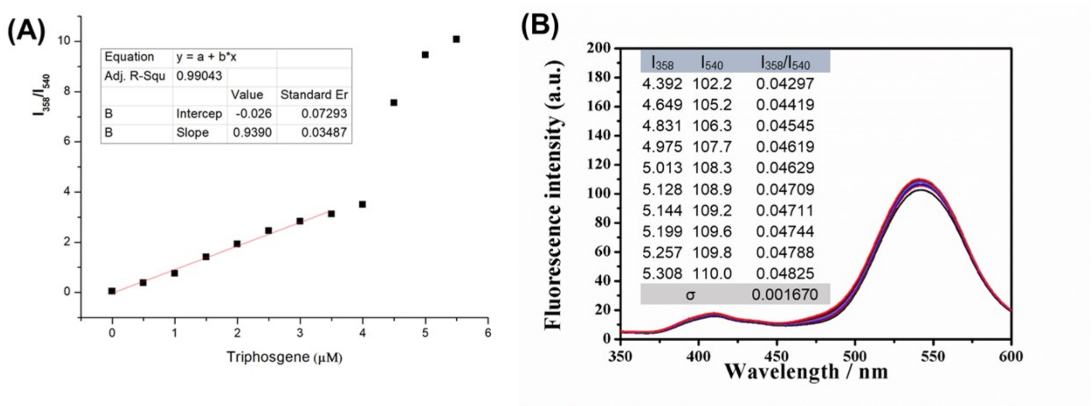

Figure S4 Measurement of the LoD for **P1** to triphosgene. (A) The ratio of the emission intensities at 358 and 540 nm *vs.* the triphosgene concentration. (B) Ten times of the blank experiment to evaluate the standard deviation σ. The triphosgene detection limit was determined to be 5.3 nM (LoD = 3σ/k, where σ is the standard deviation of the blank experiment, and k is the slope of the relationship between the emission-intensity ratio and the phosgene concentration.

5. The selectivity of P1 for the detection of phosgene.

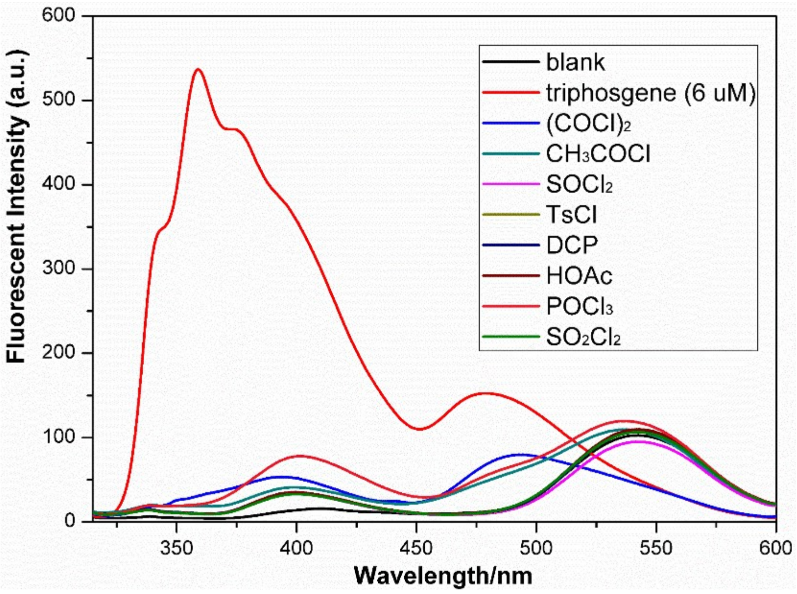

Figure S5. Fluorescence spectra of **P1** (10 μM) containing TEA (20 μL, 0.1 vol%) in 2 mL CH_2Cl_2 upon addition of phosgene or various analytes (50 μM) for 10 min. λex = 305 nm.

6. Fluorescence spectra of P1 in the presence of interfering compounds

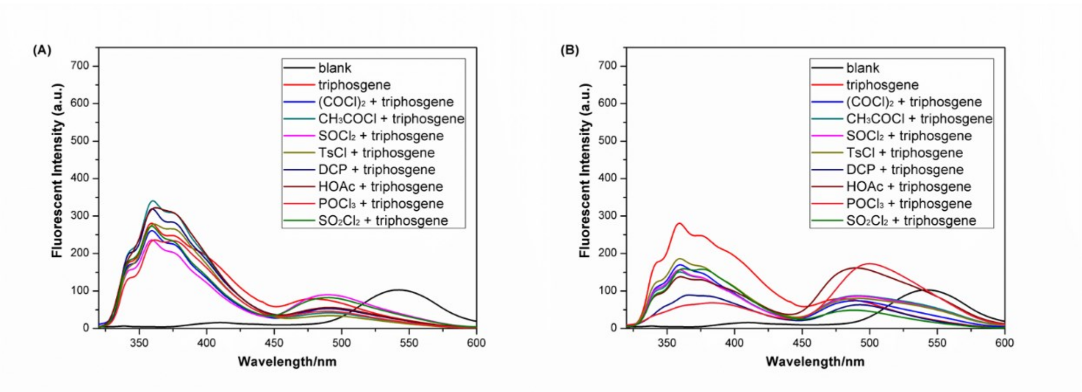

Figure S6 Fluorescence spectra of 10 μM **P1** containing TEA (20 μL, 0.1 vol%) and interferents (A, 5.0 μM; B, 10 μM) in the presence of 3.0 μM of triphosgene in 2 mL CH_2Cl_2. λex = 305 nm.

7. Table S1 Determination of phosgene in the presence of interfering compounds

Interfering compounds (5.0 μM)	Phosgene added (μM)	Phosgene found (μM)	Recovery (%)	Interfering compounds (10 μM)	Phosgene added (μM)	Phosgene found (μM)	Recovery (%)
$(COCl)_2$	9.0	8.7	96.7	$(COCl)_2$	9.0	5.15	57.1
CH_3COCl	9.0	9.8	108.9	CH_3COCl	9.0	4.7	52.0
$SOCl_2$	9.0	8.1	90.0	$SOCl_2$	9.0	4.55	50.8
TsCl	9.0	8.15	90.5	TsCl	9.0	4.75	52.8
DCP	9.0	9.45	105.2	DCP	9.0	5.6	62.4
HOAc	9.0	9.35	104.1	HOAc	9.0	2.45	27.5
$POCl_3$	9.0	7.9	87.8	$POCl_3$	9.0	4.2	46.6
SO_2Cl_2	9.0	8.15	90.7	SO_2Cl_2	9.0	1.85	20.6

8. HPLC-MS chromatogram and spectrum of the reaction mixture

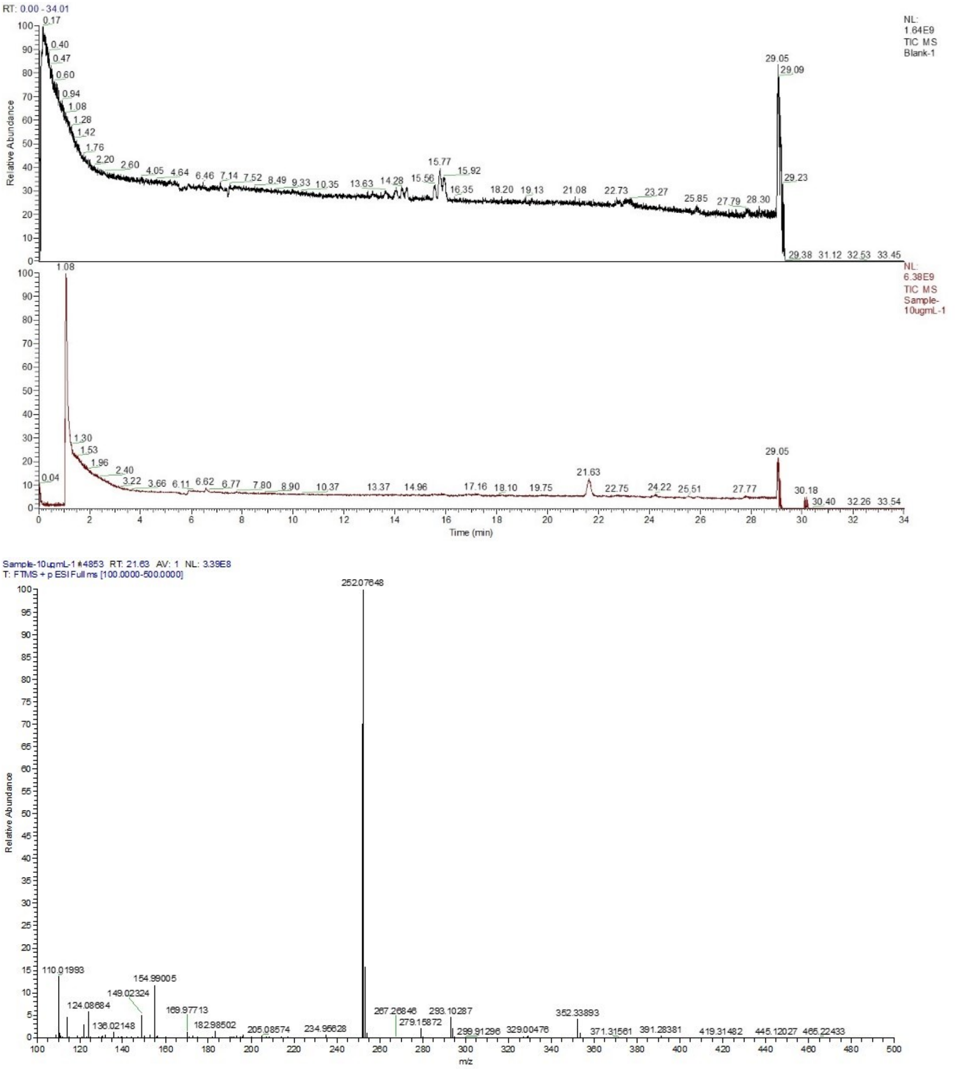

Figure S7 HPLC chromatogram (up) of the reaction mixture and MS spectrum (down) of the peak at 21.63 min. Chromatographic conditions: chromatographic column: Accucore^{TM} C18 (150 × 4.6 mm, 2.6 μm); mobile phase: 0.1% formic acid (A), 0.1% formic acid water

acetonitrile (B); gradient elution: 0 ~ 1 min, 95% A; 1~25 min, 95%~0% A; 25~29 min, 0% A; 29~29.1 min, 0%~95% A, 29.1~34 min, 95% A; flow rate: 0.2 mL/min; column temperature: 30 °C. Mass spectrometry conditions: ion source: heated electrospray ionization source (H-ESI); auxiliary gas pressure: 1.0 Mpa; capillary temperature: 300 °C, auxiliary heating temperature: 275 °C.

9. ^{1}H NMR, ^{13}C NMR and HRMS copies of P1 and P1-CO

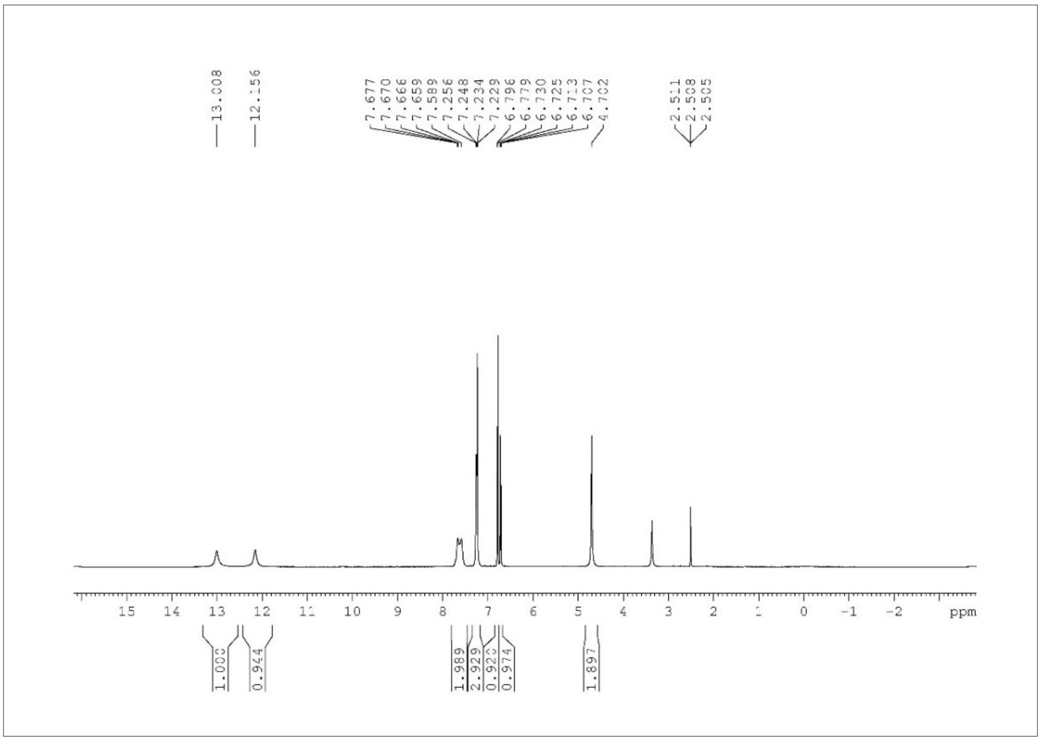

Figure S8 ^{1}H NMR of **P1**

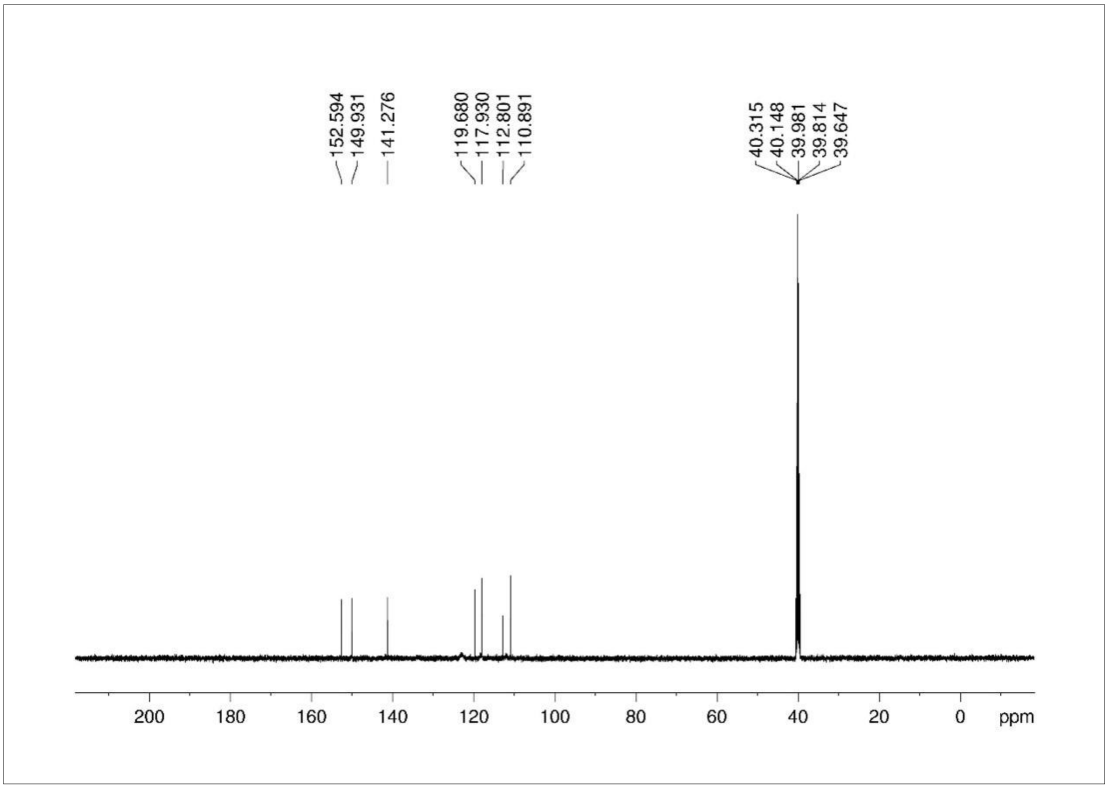

Figure S9 ^{13}C NMR of **P1**

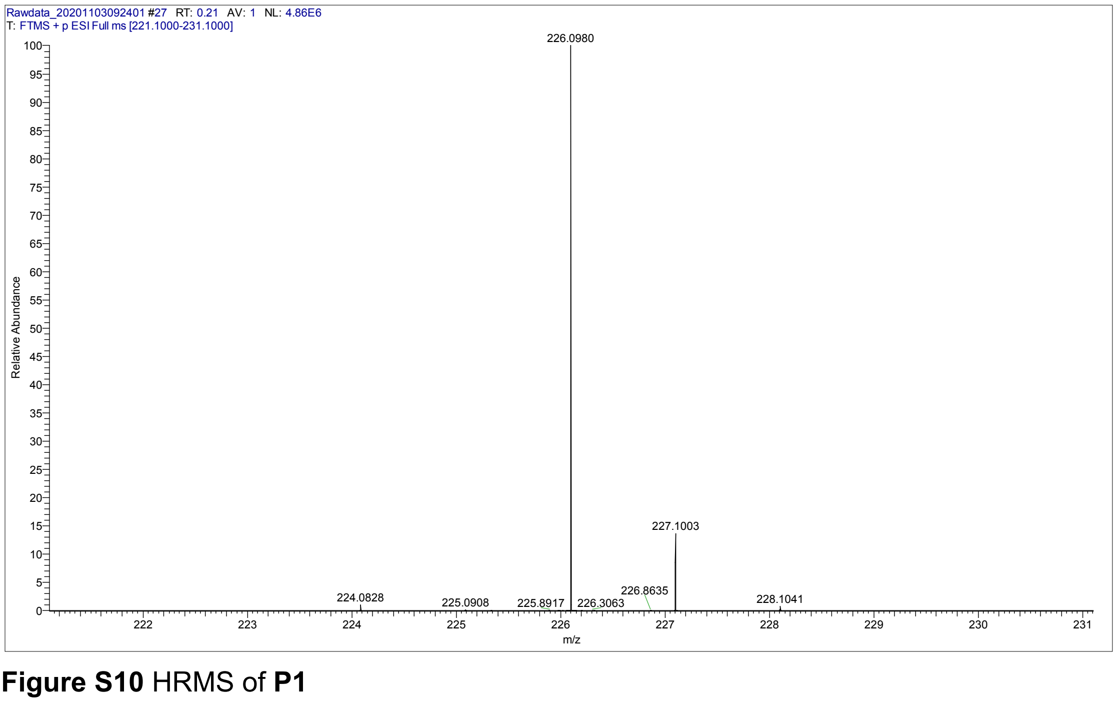

Figure S10 HRMS of **P1**

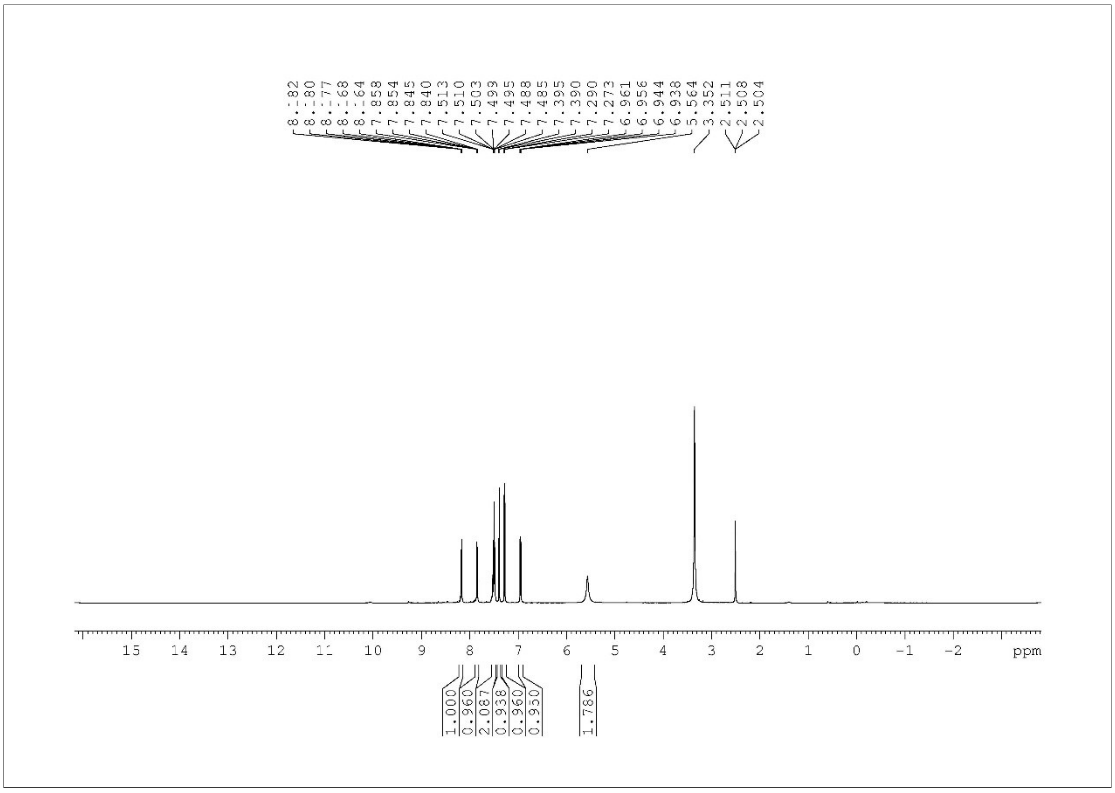

Figure S11 1HNMR of **P1-CO**

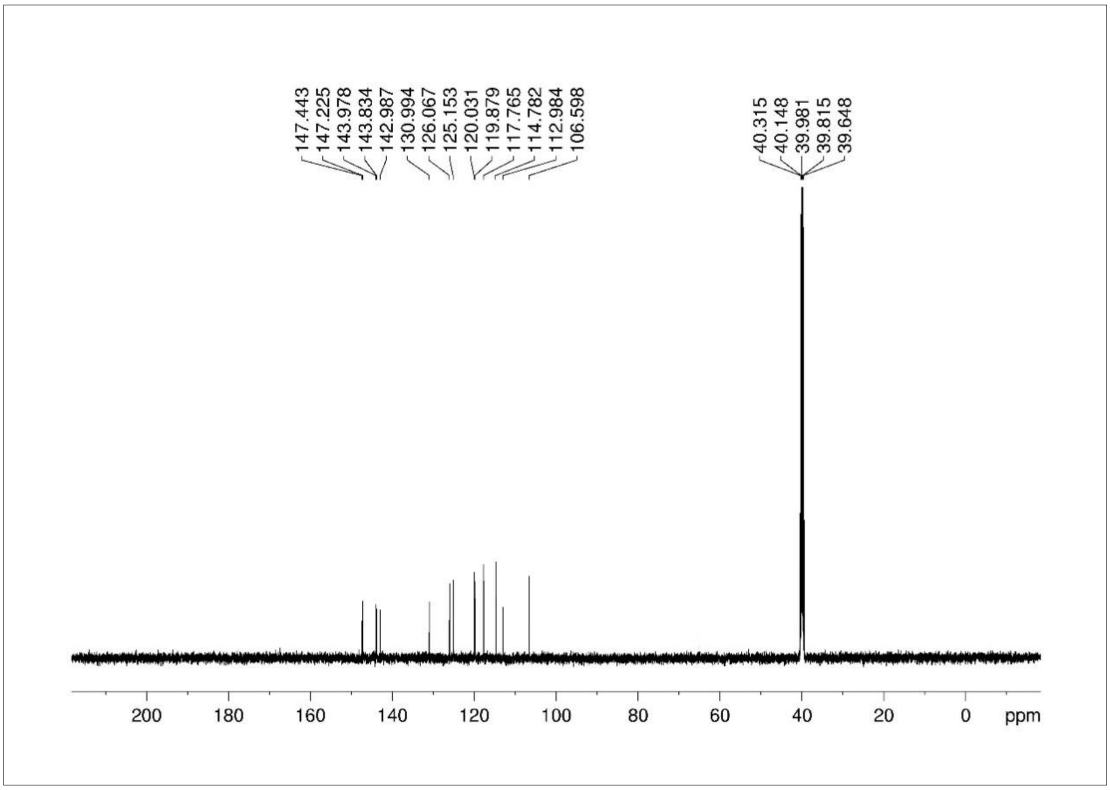

Figure S12 13C NMR of **P1-CO**

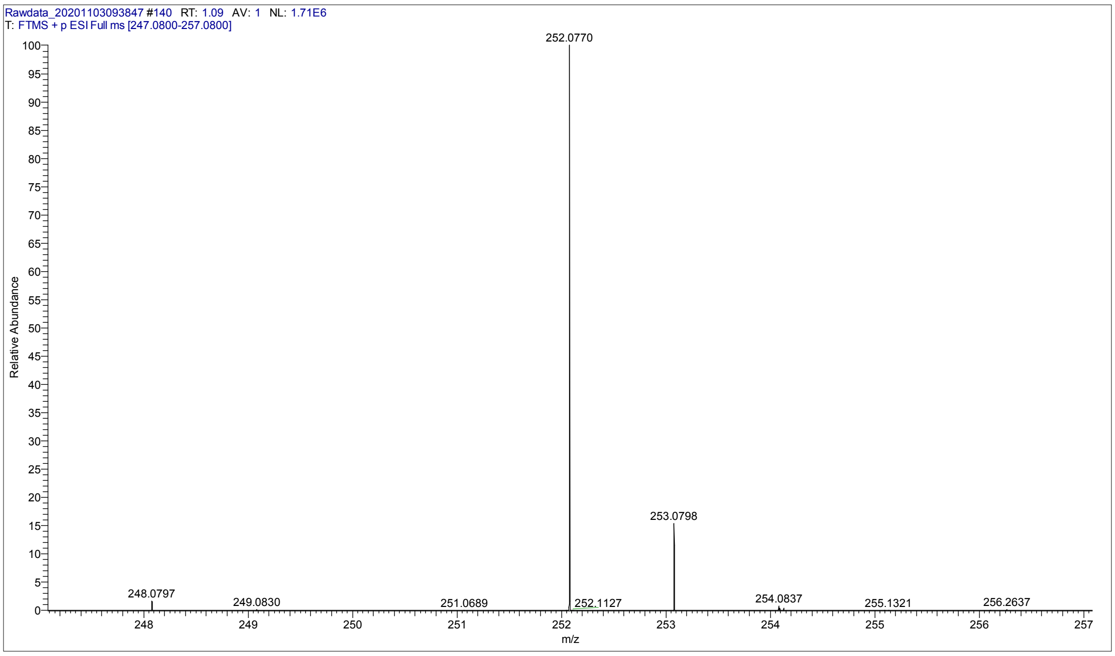

Figure S13 HRMS of **P1-CO**

10. Table S2 Comparison of recently reported probes for phosgene detection.

Structure	Mechanism	Detection type	Reaction process	λex/λem (nm)	Detection limit	Response time	Reference
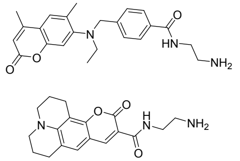	FRET	Ratiometric	intermolecular reaction of two fluorophores	λ_{ex} = 343 nm<br>λ_{em} = 464 nm	50 μM	--	*Chem. Commun.*, 2007, **12**, 1238.
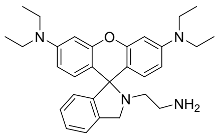	--	Turn-on	ring opening reaction of benzimidazole-fused rhodamine dye	λ_{ex} = 560 nm<br>λ_{em} = 590 nm	50 nM (Triphosgene)	20-30 s	*Chem Commun.*, 2012, **48**, 1895.
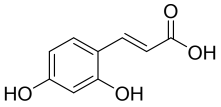	--	Turn-on	intramolecular reaction of cinnamic acids	λ_{ex} = 330 nm<br>λ_{em} = 382 nm	1 nM	--	*Anal. Chem.*, 2012, **84**, 4594
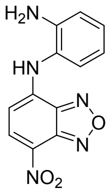	PET	Turn-on	twice carbamylation reactions	λ_{ex} = 270 nm<br>λ_{em} = 308 nm	0.7 ppb (Triphosgene)	2 min	*ACS Appl. Mater. Interfaces.*, 2016, **8**, 22246.
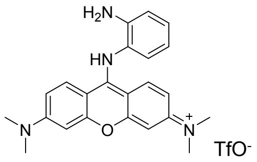	PET	Turn-on	twice carbamylation reactions	λex = 580 nm<br>λem = 593 nm	20 nM (Triphosgene)	2 min	*Angew. Chem. Int. Edit.*, 2016, **55**, 4729.

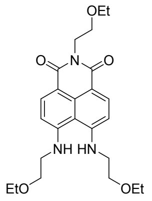	ICT	Ratiometric	twice carbamylation reactions	λex = 410 nm<br>λem= 511/442 nm	1.3 nM<br>(Triphosgene)	20 min	*Chem. Commun.*, 2017, **53**, 1530.
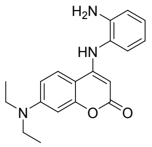	PET	Turn-on	twice carbamylation reactions	λex = 368 nm<br>λem = 446 nm	3 nM<br>(Triphosgene)	0.5 min	*ACS Sens.*, 2017, **2**, 178.
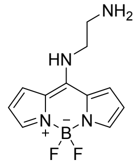	ICT	Ratiometric	twice carbamylation reactions	λ_{ex} = 390/465 nm<br>λ_{em} = 445/512 nm	0.12 nM<br>(Phosgene)	1.5 s	*ACS Appl. Mater. Interfaces*, 2017, **9**, 13920.
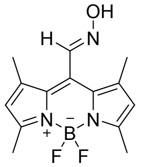	--	Turn-on	Dehydration of oxime to nitrile	λ_{ex} = 530 nm<br>λ_{em} = 570 nm	0.09 ppb<br>(Triphosgene)	10 s	*Anal. Chem.*, 2017, **89**, 12837.
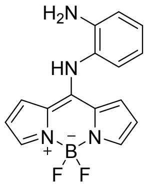	PET	Turn-on	twice carbamylation reactions	λ_{ex} = 450 nm<br>λ_{em} = 530 nm	2.7 nM<br>(Triphosgene)	15 s	*Anal. Chem.*, 2017, **89**, 4192.

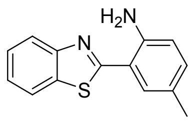	ESIPT	Ratiometric	twice carbamylation reactions	λ_{ex} = 375 nm<br>λ_{em} = 445/495 nm	0.14 ppm (Phosgene)	5 min	*Anal. Chem.*, 2017, **89**, 12596.
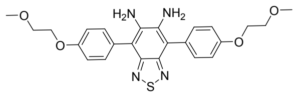	ICT	Turn-on	twice carbamylation reactions	λ_{ex} = 380 nm<br>λ_{em} = 508 nm	20 nM (Phosgene)	20 min	*Org. Chem. Front.*, 2017, **4**, 1719.
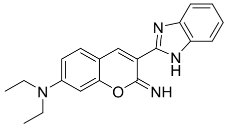	ICT	Ratiometric	twice carbamylation reactions	λ_{ex} = 440 nm<br>λ_{em} = 482/550 nm	27 nM (Phosgene)	2 min	*Anal. Chim. Acta.*, 2018, **1029**, 97.
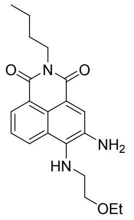	ICT	Turn-on	twice carbamylation reactions	λex = 400 nm<br>λem = 468 nm	0.2 nM (Triphosgene)	30 s	*Chem. Eur. J.*, 2018, **24**, 5652.
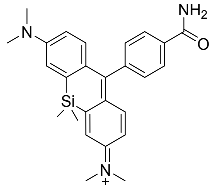	--	Turn-on	conversion of amide to nitrile	λex = 653 nm<br>λem = 679 nm	8.9 nM (Triphosgene)	4 min	*J. Mater. Chem. C.*, 2018, **6**, 10472.
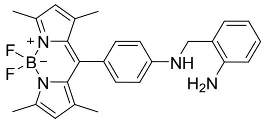	PET	Turn-on	twice carbamylation reactions	λex = 460 nm<br>λem = 511 nm	179 nM (Triphosgene)	10 s	*Chem. Eur. J.*, 2018, **24**, 3136.

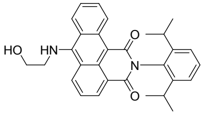	ICT	Ratiometric	twice carbamylation reactions	λ_{ex} = 434/502 nm λ_{em} = 482/615 nm	2.3 nM (Phosgene)	5 min	*Anal. Chem.*, 2018, **90**, 8686.
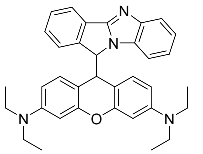	--	Turn-on	Spirocyclic ring -open reaction	λ_{ex} = 530 nm λ_{em} = 578 nm	3.2 ppb (Triphosgene)	2 min	*Anal. Chem.*, 2018, **90**, 3382.
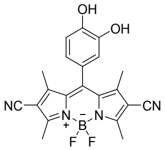	PET	Turn-on	twice carbamylation reactions	λ_{ex} = 480 nm λ_{em} = 516 nm	24 pM (Phosgene)	within 3 s	*Sens. Actuator B-Chem.*, 2019, **283**, 458.
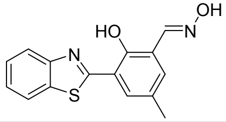	ESIPT	Turn-on	conversion of oxime to nitrile	λ_{ex} = 438 nm λ_{em} = 474 nm	0.48 nM (Phosgene)	20 min	*Dyes Pigment.*, 2019, **163**, 483.
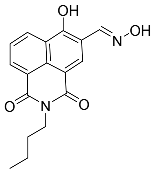	ESIPT	Ratiometric	conversion of oxime to isoxazole	λ_{ex} = 382 nm λ_{em} = 495/577nm	0.087 ppm (Phosgene)	1.43 s	*J. Mater. Chem. A.*, 2019, **7**, 1756.
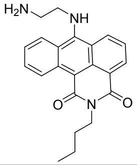	ICT	Ratiometric	twice carbamylation reactions	λ_{ex} = 470 nm λ_{em} = 520/610 nm	0.09 nM (Phosgene)	Within 20 s	*Dyes Pigment.*, 2019, **163**, 489.

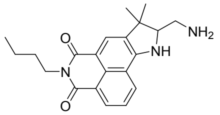	ICT	Ratiometric	twice carbamylation reactions	λ_{ex} = 400 nm<br>λ_{em} = 488/548 nm	0.3 nM (Phosgene)	60 s	*J. Mater. Chem. C.*, 2019, **7**, 1510.
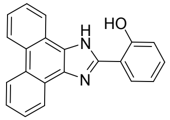	ESIPT	Ratiometric	twice carbamylation reactions	λ_{ex} = 335 nm<br>λ_{em} = 393/469 nm	0.14 ppm (Phosgene)	30 s	*Talanta*, 2019, **200**, 78.
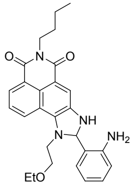	ICT	Turn-on	twice carbamylation reactions	λ_{ex} = 390 nm<br>λ_{em} = 422/526 nm	3.2 nM (Phosgene)	within 10 s	*Anal Chem.*, 2019, **91**, 5690.
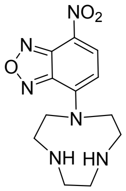	PET	Turn-on	twice carbamylation reactions	λ_{ex} = 460 nm<br>λ_{em} = 525 nm	1.2 nM (Triphosgene)	within 20 s	*Anal. Methods*, 2019, **11**, 4600.
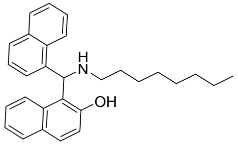	PET	Turn-on	twice carbamylation reactions	λ_{ex} = 350 nm<br>λ_{em} = 430 nm	0.4 μM (Triphosgene)	20 s	*New J. Chem.*, 2019, **43**, 11743.
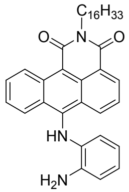	PET	Turn-on	twice carbamylation reactions	λ_{ex} = 440 nm<br>λ_{em} = 500 nm	72 nM (Phosgene)	2 min	*Anal Chem.*, 2019, **91**, 12070.

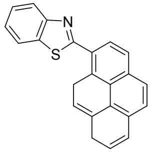	PET	Ratiometric	chloroformylation reaction of N atom	λ_{ex} = 400 nm<br>λ_{em} = 422/445/477 nm	1.54 nM (Phosgene)	within 50 s	*New J. Chem.*, 2019, **43**, 14991.
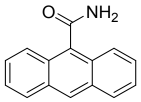	--	Turn-on	Conversion of amide to nitrile	λ_{ex} = 342 nm<br>λ_{em} = 440 nm	5.56 nM (Phosgene)	1.4 min	*ChemistrySelect*, 2019, **4**, 2968.
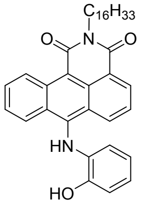	PET	Turn-on	twice carbamylation reactions	λ_{ex} = 432nm<br>λ_{em} = 484nm	4.6 nM (Phosgene)	15 s	*Chem. Commun.*, 2019, **55**, 13753.
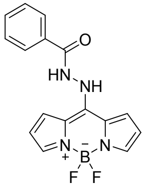	PET	Turn-on	twice carbamylation reactions	λ_{ex} = 467 nm<br>λ_{em} = 530 nm	0.15 nM (Phosgene)	1.5 s	*Org. Lett.*, 2019, **21**, 9497.
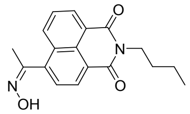	ICT	Turn-on	Beckmann rearrangement of ketoxime	λ_{ex} = 367 nm<br>λ_{em} = 448 nm	6.3 nM (Phosgene)	15 min	*Dyes Pigment.*, 2020, **173**, 107854.
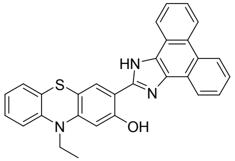	--	Turn-on	twice carbamylation reactions	λ_{ex} = 440 nm<br>λ_{em} = 492 nm	84.2 nM (Phosgene)	2 min	*Dyes Pigment.*, 2020, **173**, 107933.

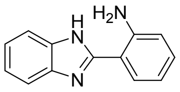	--	Ratiometric	twice carbamylation reactions	λ_{ex} = 370 nm<br>λ_{em} = 416 nm	1.27 nM (Phosgene)	1 min	*Talanta*, 2021, **221**, 121477.
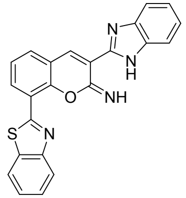	ICT	Turn-on	twice carbamylation reactions	λex = 390 nm<br>λem = 442/483/517 nm	1.65 nM (Phosgene)	3 min	*Sens. Actuator B-Chem.*, 2021, **326**, 128837.
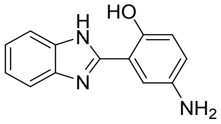<br>**This work**	ESIPT	Ratiometric	twice carbamylation reactions	λex = 305 nm<br>λem = 358/540 nm	5.3 nM (Trihosgene)	Within 50 s	
